# One year duration of immunity of a combination *Bordetella bronchiseptica* - canine parainfluenza oral vaccine in dogs

**DOI:** 10.3389/fvets.2025.1634190

**Published:** 2025-10-14

**Authors:** Sarah A. Wiechert-Brown, Haley M. Classe, Jennifer C. Dant, Rhonda L. LaFleur, Zach Xu, Ian Tarpey

**Affiliations:** ^1^Research and Development Department, Merck Animal Health, Elkhorn, NE, United States; ^2^Research and Development Department, MSD Animal Health, Boxmeer, Netherlands

**Keywords:** canine, parainfluenza, *Bordetella bronchiseptica*, oral, vaccination

## Abstract

**Introduction:**

Canine infectious respiratory disease complex (CIRDC), widely known as kennel cough or infectious tracheobronchitis, is a highly contagious disease in dogs caused by multiple bacterial and viral pathogens. Two significant pathogens that contribute to CIRDC are *Bordetella bronchiseptica (B. bronchiseptica)* and canine parainfluenza virus (CPI). Recently, the first oral, modified live, vaccine to contain a combination of these two pathogens has become commercially available.

**Methods:**

To evaluate the efficacy of both fractions of this vaccine, duration of immunity studies were conducted in six- to eight-week-old beagles. In both studies, dogs were randomized into two treatment groups and vaccinated once by the oral route with either test vaccine or placebo vaccine. In the CPI study, dogs were challenged with virulent CPI one year after vaccination and observed daily for 14 days post-challenge. Nasal swabs were also collected daily for 10 days post-challenge to evaluate the shedding of the challenge virus. In the *B. bronchiseptica* study, dogs were challenged with virulent *B. bronchiseptica* one year after vaccination and observed twice daily for 28 days post-challenge for clinical signs of disease.

**Results:**

The results from these studies demonstrated a significant reduction in nasal shedding of CPI (0.2 log_10_FAID_50_/mL in test-vaccinated compared to 1.1 log_10_FAID_50_/mL in placebo-vaccinated dogs) as well as a significant reduction in clinical signs associated with *B. bronchiseptica* (9% of test-vaccinated compared to 74% of placebo-vaccinated dogs).

**Conclusion:**

Taken together, these studies demonstrated that oral vaccination with a combination vaccine containing *B. bronchiseptica* and CPI is effective at preventing disease for at least one year following vaccination.

## 1 Introduction

Canine infectious respiratory disease complex (CIRDC) is an upper respiratory tract disease caused by several different bacterial and viral pathogens either alone or in combination (1). *Bordetella bronchiseptica* (*B. bronchiseptica*), as well as canine parainfluenza virus (CPI), have been identified as important pathogens implicated in CIRDC pathogenesis, and outbreaks caused by CPI (1–3) and *B. bronchiseptica* (4, 5) have been reported worldwide. CPI is transmitted via airborne particles for up to 2 weeks post-infection, while *B. bronchiseptica* is transmitted either by direct contact or via airborne particles for up to several months post-infection (6). Consequently, CIRDC spreads quickly among dogs housed together, particularly in shelters and kennels, and can continue infecting dogs for weeks to months (7).

Dogs infected with CPI may or may not exhibit clinical signs of disease including a dry, harsh cough with or without fever and nasal discharge up to 7 days (6), and these clinical signs can progress in severity during coinfections (8). *B. bronchiseptica* infection in dogs may induce pyrexia and cause a dry hacking cough with retching and nasal discharge, progressing to pneumonia in severe cases (6). CPI has been shown to suppress the innate branch of the immune system through the destruction of cilia and ciliated epithelium increasing the likelihood of coinfections to occur and thus, the severity of disease (9). Furthermore, *B. bronchiseptica* can suppress both the innate and adaptive immune responses through its virulence mechanisms which enable production of anti-inflammatory mediators (10). These factors, combined with the vulnerability of the respiratory innate immune defense, make coinfections with these two pathogens a significant challenge to the immune system.

Interestingly, CPI and *B. bronchiseptica* are two of the most frequently isolated pathogens identified in the nose and oropharynx from dogs with upper respiratory infections, making them ideal vaccine targets (11, 12). Currently, there are several licensed oral vaccines effective against *B. bronchiseptica* and one has a reported duration of immunity (DOI) of 13 months, but none of the currently licensed oral vaccines contain CPI (13). The current studies were conducted to evaluate the effectiveness of a bivalent, oral vaccine (*B. bronchiseptica* + CPI) when administered as a single dose to dogs who were then challenged with either virulent CPI or virulent *B. bronchiseptica* 1 year following vaccination.

## 2 Materials and methods

### 2.1 Ethics statement

All enrolled dogs were handled in compliance with Merck Animal Health Institutional Animal Care and Use Committee (IACUC) guidelines and approval was obtained prior to study initiation (protocols E-2020-64 and E-2021-45).

### 2.2 Vaccine formulations

For vaccine preparation, the CPI strain was grown in Vero cells at 37 °C ± 2 °C for 48–144 h, and the *B. bronchiseptica* strain was grown in a fermenter, harvested, and concentrated. The CPI and *B. bronchiseptica* antigens were combined with stabilizer (hydrolyzed gelatin, N-Z Amine, sorbitol, and sodium phosphate, dibasic), HEPES (4-(2-hydroxyethyl)-1-piperazineethanesulfonic acid) and sterile water, lyophilized, and then reconstituted with 1.0 mL of sterile water prior to use. In the CPI DOI study, the CPI fraction was at minimum protective dose (MPD) (> 6.0 log_10_HAID_50_/dose) (Hemadsorption Antibody Infectious Dose), and the *B. bronchiseptica* fraction was at field dose (> 1.0 × 10^8^ cfu/dose). In the *B. bronchiseptica* DOI study, the *B. bronchiseptica* fraction was at MPD (> 1.0 × 10^7^ cfu/dose), and the CPI fraction was at field dose (≥10-fold higher than the MPD). The placebo vaccines consisted of all components of the test vaccine except the antigen being evaluated. The test vaccine is commercially available as Nobivac® Intra-Trac® Oral BbPi.

### 2.3 Challenge materials

The heterologous CPI strain was grown on primary dog kidney cells to a titer of > 5.0 log_10_FAID_50_/mL and frozen at < −50 °C. On the day of challenge, the frozen challenge virus was thawed, and 1.4 mL was administered to the upper respiratory tract of each dog. The challenge dose was optimized in preliminary studies to induce CPI viral shedding in at least 50% of the animals.

The heterologous *B. bronchiseptica* strain was grown on tryptic soy with 5% sheep blood agar plates. The plates were incubated for approximately 18 h at 36 ± 2 °C, and the pure bacterial growth was washed off the agar plates with tryptose phosphate broth and centrifuged. The pellet was adjusted to > 1.0 × 10^8^ cfu/mL, and 1.0 mL was administered by the intranasal route. The challenge dose was optimized in preliminary studies to generate clinical signs of disease in at least 50% of the animals.

### 2.4 Study design

Six- to eight-week-old, purpose-bred beagles that had not been exposed to or vaccinated for *B. bronchiseptica* or CPI were enrolled in the two studies. Dogs were housed in an isolation facility in barrier rooms and randomized into two treatment groups using housing units and litter as randomization factors for the CPI study or using pre-vaccination antibody titers and litter as randomization factors for the *B. bronchiseptica* study. The dogs were separated by treatment group prior to vaccination and remained as such until challenge when they were comingled to ensure an equal number of vaccinated dogs and placebo-vaccinated control dogs were housed in the same room. All dogs were vaccinated orally once (study day 0) with a 1 mL dose of the *B. bronchiseptica* + CPI vaccine (*n* = 23) or placebo vaccine (*n* = 23) using the Immuno-Mist-R™ applicator attached to a 3-mL Luer-Lok™ syringe for each study. Prior to vaccination, whole blood for serum and nasal swabs were collected. Following vaccination, whole blood for serum was collected monthly until the dogs were challenged, and nasal swabs were collected just prior to challenge.

In the CPI study, 23 test-vaccinated and 23 placebo-vaccinated control dogs were challenged with CPI via the upper respiratory tract 12 months following vaccination. During the 14-day post-challenge observation period, clinical signs including nasal and ocular discharge, coughing, sneezing, depression, fever (defined as ≥103.5° F), and dyspnea were monitored daily, and nasal swabs were collected daily for 10 days.

In the *B. bronchiseptica* study, 22 test-vaccinated dogs and 23 placebo-vaccinated control dogs were challenged by the intranasal route 13 months following vaccination with *B. bronchiseptica.* One dog from the test vaccine treatment group was euthanized prior to challenge due to aggressive behavior that raised animal welfare concerns as well as safety concerns for animal care personnel. During the 28-day post-challenge observation period, clinical signs including coughing, nasal discharge, depression, and dyspnea were monitored for 30 min each day, twice daily, and nasal swabs were collected twice a week.

### 2.5 Canine parainfluenza virus serum neutralization assay

Whole blood collected into serum separation tubes (BD; Franklin Lakes, NJ) was allowed to clot for a minimum of 2 h at 15–30 °C and then centrifuged at 1000 × *g* to separate the serum. Serum samples were tested in a serum neutralization assay to evaluate the serological response post-vaccination. Briefly, each serum sample was serially diluted and incubated with CPI for 30–60 min before inoculated onto a confluent monolayer of dog kidney cells planted on 96-well tissue culture plates (Falcon; Corning, NY). Plates were incubated at 36 ± 2 °C with 4–6% CO_2_ for 7 days. Monolayers were then fixed and stained with fluorescein-conjugated CPI antiserum (VMRD; Pullman, WA), and titers were calculated using the Spearman-Karber method.

### 2.6 Canine parainfluenza virus titration for viral shedding

Polyester fiber-tipped swabs (Fisherbrand; Pittsburgh, PA) were wetted in transport media [Dulbecco’s Modified Eagles Medium (Corning; Corning, NY), gentamicin (Gibco; Grand Island, NY), and amphotericin-B (Corning; Corning, NY)] and inserted into each nostril of the dog. In the laboratory, swabs were removed from the transport media, and the liquid contents were centrifuged at 500 × *g*. The supernatant was used to inoculate a confluent monolayer of dog kidney cells that were planted on 96-well tissue culture plates (Falcon; Corning, NY). Plates were incubated at 36 ± 2 °C for 7 days, fixed with 80% acetone (Fisher Scientific, Pittsburg, PA), and stained with fluorescein-conjugated CPI antiserum (VMRD; Pullman, WA). Virus titers were calculated using the Spearman-Karber method.

### 2.7 *B. bronchiseptica* agglutination assay

Whole blood collected into serum separation tubes (BD; Franklin Lakes, NJ) was allowed to clot for a minimum of 2 h at 15–30 °C and then was centrifuged at 1000 × *g* to separate the serum. Serum samples were tested in an agglutination assay to evaluate the serological response post-vaccination. Briefly, each serum sample (along with a positive and negative control sample) was serially diluted 2-fold in a U-bottom microtiter plate (Nunc; Rochester, NY) using normal saline containing 0.1% gelatin as the diluent. *B. bronchiseptica* antigen was added to each well and mixed for 15–30 s on a microtiter plate shaker (Lab-Line Instruments, Inc.; Melrose Park, IL; Model 4,625). The plates were incubated at 36 ± 2 °C for 1–3 h and then incubated at 2–7 °C for 36–72 h until they were visually assessed for agglutination. The titers were expressed as the reciprocal of the highest dilution showing complete agglutination.

### 2.8 *B. bronchiseptica* titration for bacterial shedding

Two calcium alginate swabs (McKesson; Irving, TX) were used to collect specimens from each nostril using one swab per nostril and placed in transport media [Tryptose phosphate broth (BD; Franklin Lakes, NJ) with 10% glycerol (Fisher; Pittsburgh, PA)]. In the laboratory, swabs were removed from the transport media, and 10-fold serial dilutions of the nasal swab material were performed in tryptose phosphate broth. Each dilution that was expected to yield 30–300 colonies (0.1 mL/plate) was spread onto three replicate MacConkey agar plates (Thermo Scientific; Waltham, MA). The plates were incubated at 36 ± 2 °C for 48–96 h, colonies counted, and results recorded (cfu/mL).

### 2.9 Statistical analysis

Statistical analysis for duration of CPI shedding was performed using R software version 3.5.0. Statistical significance for serologic geometric means and viral load were performed using a two-tailed Student’s T-test assuming equal variances in Microsoft® Excel® version Microsoft 365® and significance was declared for *p*-values < 0.05.

An affected dog in the *B. bronchiseptica* study was defined as having spontaneous coughing or spontaneous coughing with retching on 2 or more consecutive days during the post-challenge observation period. The proportion of affected dogs in the vaccinated and placebo-vaccinated control group was calculated and compared using Fisher’s Exact Test. Statistical significance for serologic geometric means and bacterial load were calculated and compared using a two-tailed Welch’s T-test in Microsoft® Excel® version Microsoft 365®. Significance was declared for *p*-values < 0.05.

## 3 Results

### 3.1 Canine parainfluenza virus serology

**Figure 1:**
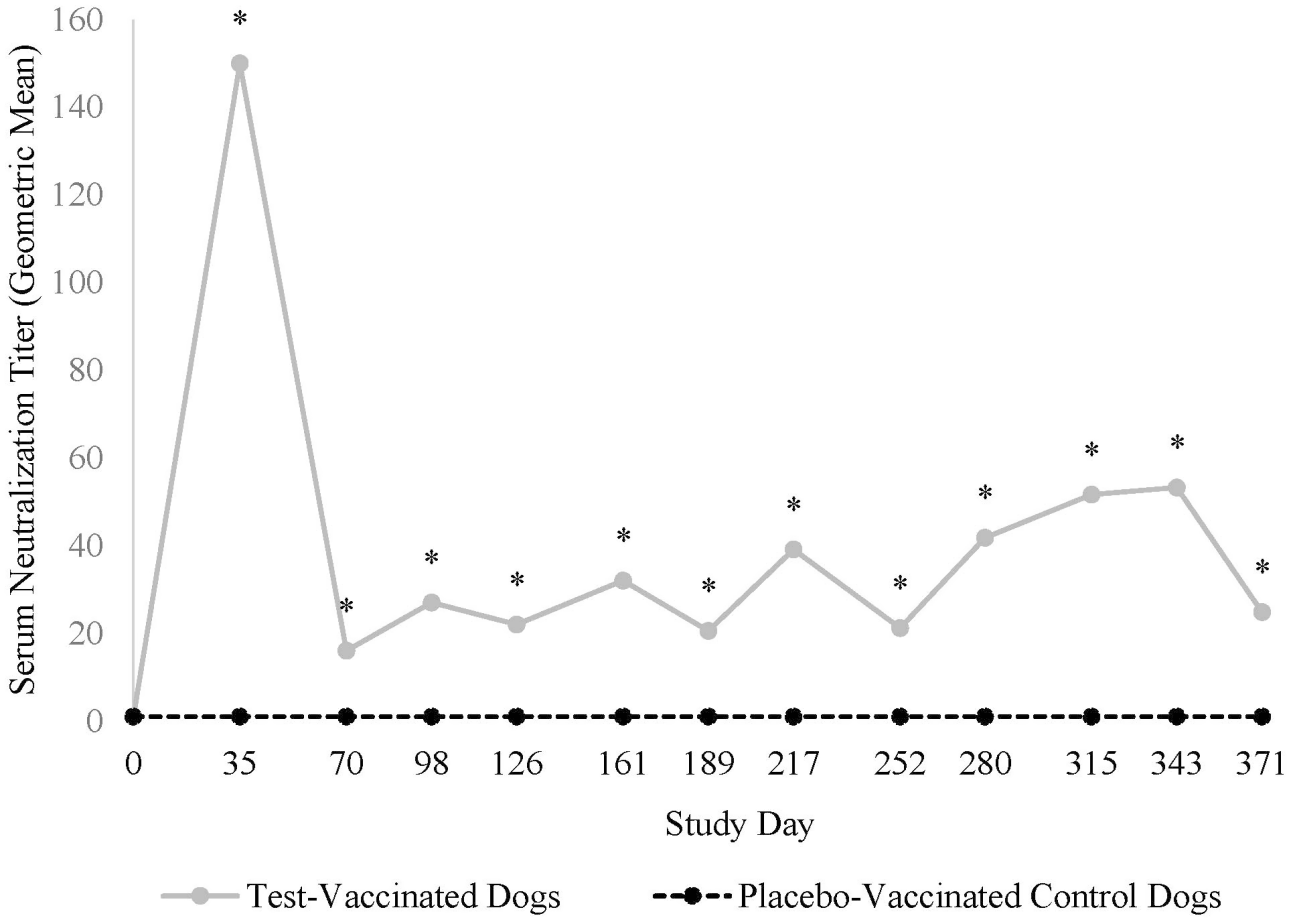
Canine parainfluenza virus serology. The graph displays the geometric mean CPI serum neutralization titers throughout the 12-month vaccination period. CPI serum neutralization titers peaked 35 days post-vaccination and then decreased to a level that remained constant throughout the 1-year post-vaccination period (*p < 0.01).

All dogs were seronegative (<2) for antibodies against CPI prior to vaccination, indicating no prior exposure. Vaccination with the placebo vaccine did not induce antibodies specific to CPI. In contrast, vaccination with the test vaccine induced CPI serum neutralization titers of ≥ 23 in 21 (91%) of 23 test-vaccinated dogs, with peak titers ranging from <2 to 2,435 (GMT = 150) 5 weeks after vaccination (Figure 1). Titers decreased thereafter and remained constant throughout the 12-month vaccination period. CPI serum neutralization titers were significantly higher in the test-vaccinated dogs compared to the placebo-vaccinated control dogs at all time points tested (study days 35, 70, 98, 126, 161, 189, 217, 252, 280, 315, 343, and 371; *p*-values < 0.01).

### 3.2 Nasal shedding of canine parainfluenza virus

**Figure 2:**
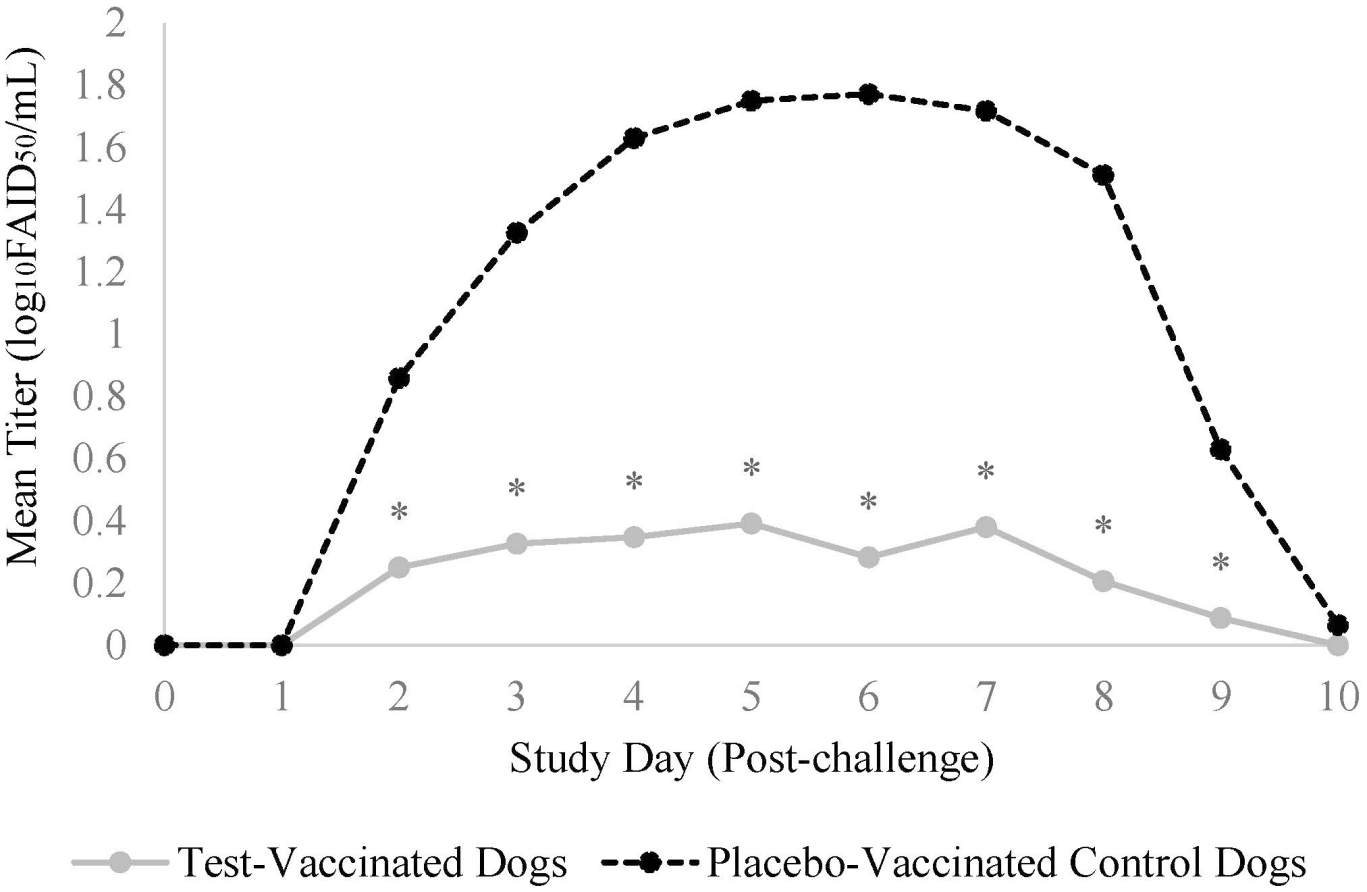
Nasal shedding of canine parainfluenza virus. The graph displays the mean titer of CPI virus shed from the nose for 10-days post-challenge. Nasal swabs were collected daily for 10 days post-challenge and titrated for CPI virus. The daily mean titer for each treatment group was calculated and compared. The test vaccine significantly reduced the amount of CPI virus that was shed from dogs on days 2-9 following challenge (*p ≤ 0.04).

CPI was not isolated from nasal swabs prior to challenge, but 2 days following challenge, dogs from both treatment groups started shedding CPI (Figure 2). In total, 22 (96%) of the 23 placebo-vaccinated control dogs shed CPI, compared to only 13 (57%) of the 23 test-vaccinated dogs (data not shown). Test-vaccinated dogs averaged 0.2 log_10_FAID_50_/mL of CPI shed during the post-challenge phase (*p*-value = 0.0008) compared to 1.1 log_10_FAID_50_/mL of CPI shed from the placebo-vaccinated control dogs. Specifically, the vaccinated treatment group demonstrated significant reduction in viral load on study days 2–9 (Figure 2, *p*-values ≤ 0.04).

**Figure 3:**
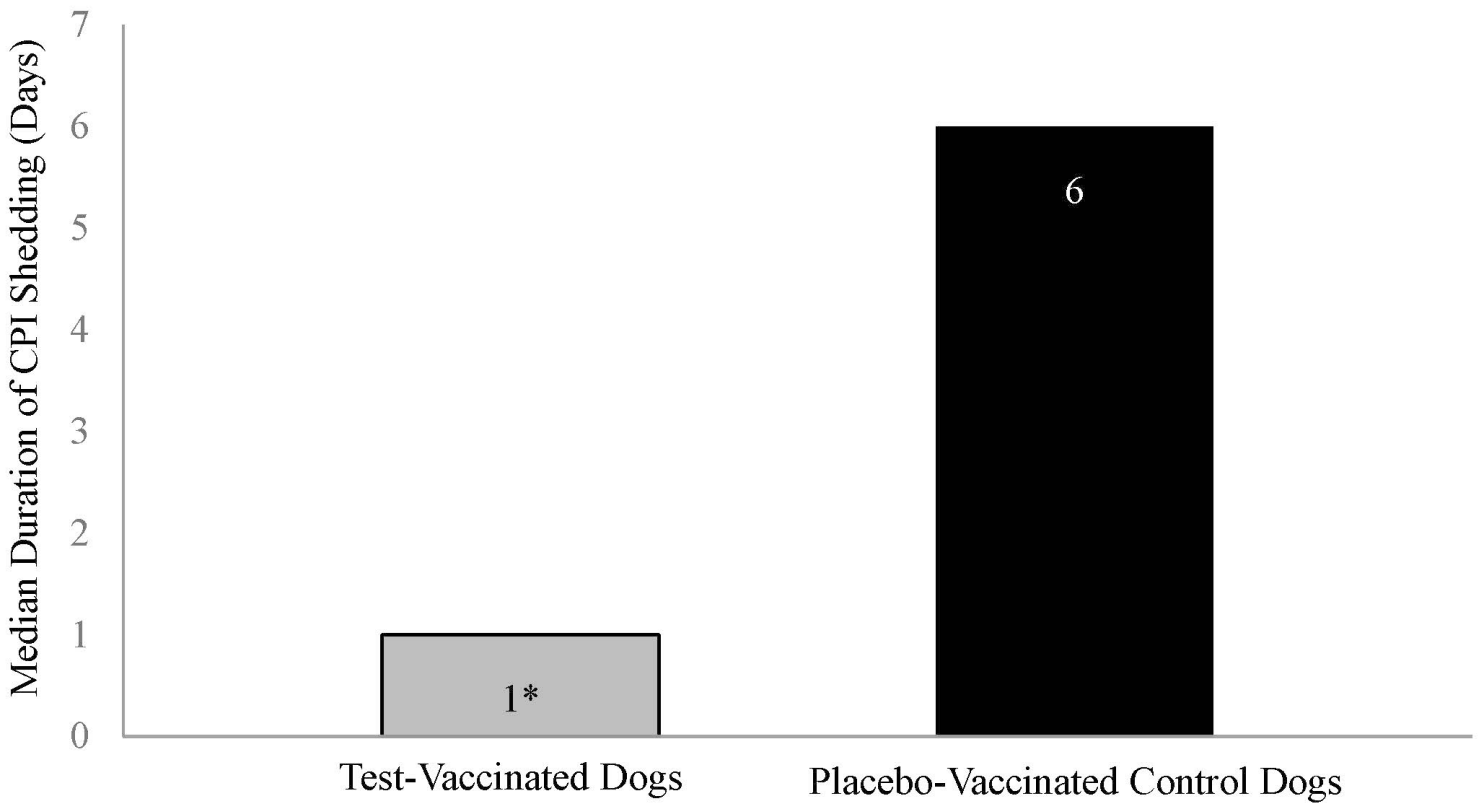
Duration of canine parainfluenza virus nasal shedding. The graph displays the median duration of CPI shedding by each treatment group. Duration of shedding, from first to last occurrence, was calculated for each dog, and the median number of days determined. The test vaccine significantly reduced the duration of CPI viral shedding in dogs following challenge (*p = 0.001).

Duration of virus shedding post-challenge was the primary variable to evaluate vaccine efficacy and was defined as the first through the last day that CPI was isolated from nasal swabs. The median duration of shedding for the placebo-vaccinated control dogs was 6 days, which was significantly more than the 1 day for the vaccinated dogs (Figure 3, *p*-value = 0.001).

### 3.3 Clinical signs of canine parainfluenza virus

Consistent with previous laboratory CPI challenge studies (14, 15), there were very few clinical signs of disease during the post-challenge period. None of the placebo-vaccinated control dogs showed clinical signs of a CPI infection, and only 1 of the test-vaccinated dogs spontaneously coughed on two separate days.

### 3.4 *B. bronchiseptica* serology

**Figure 4:**
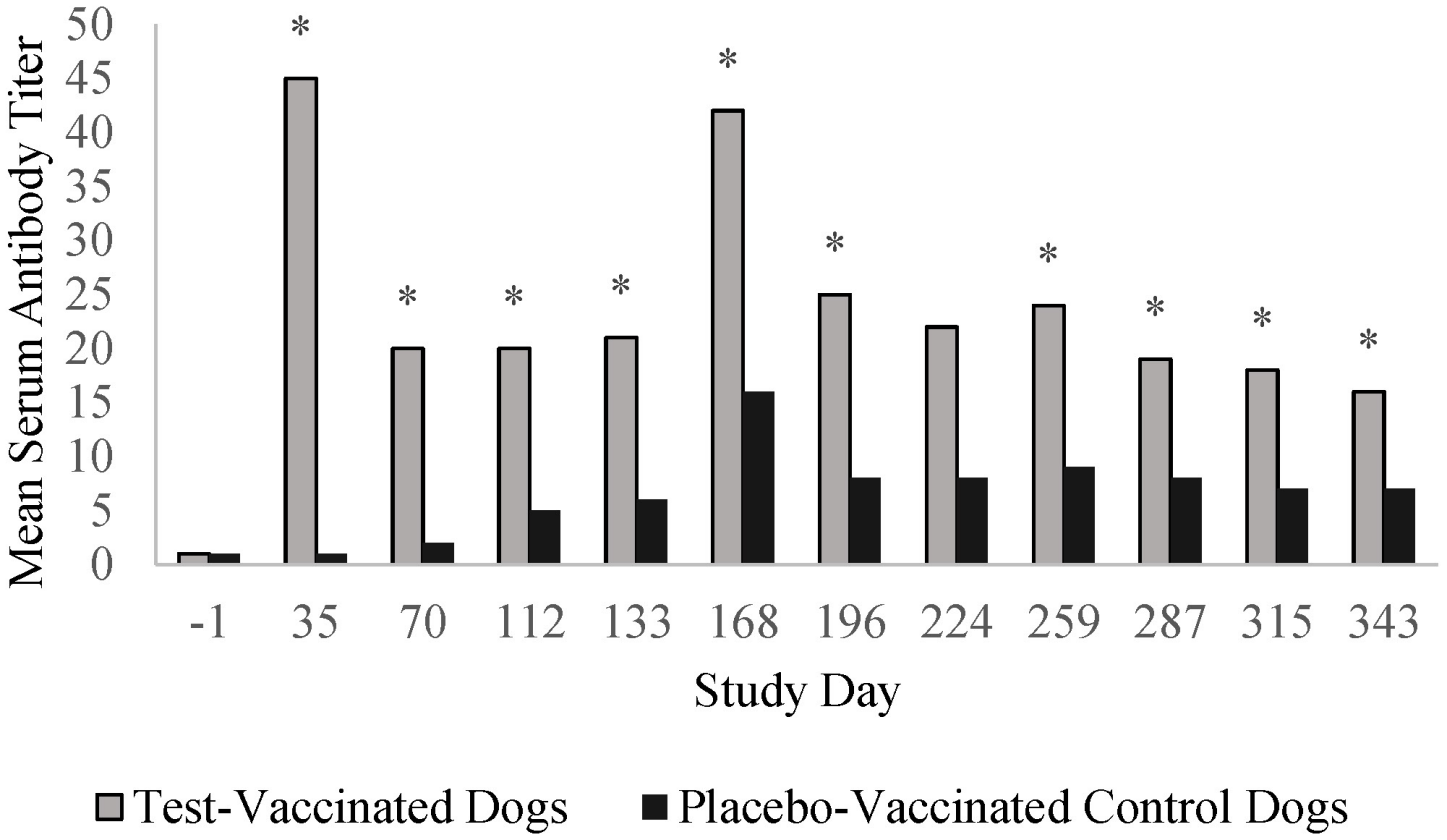
*Bordetella bronchiseptica* serology. The graph displays the mean *B. bronchiseptica* serum antibody titers throughout the 13-month vaccination period. *B. bronchiseptica* serum antibody titers peaked 35 days post-vaccination and then decreased to a level that remained relatively constant throughout the 1-year post-vaccination period (*p < 0.05).

Prior to vaccination, all dogs in the *B. bronchiseptica* study had low IgG antibody titers (≤ 4) to *B. bronchiseptica*. Vaccination with the placebo vaccine did not induce high IgG antibody titers specific to *B. bronchiseptica*. In contrast, vaccination with the test vaccine induced titers ≥ 16 in all of the vaccinated dogs, with peak titers ranging from 16 to 128 (GMT = 45) 5 weeks after vaccination (Figure 4). Titers decreased thereafter and remained relatively constant throughout the 13-month vaccination period. Serum antibody titers were significantly higher in the test-vaccinated dogs compared to the placebo-vaccinated control dogs at all time points tested (study days 35, 70, 112, 133, 168, 196, 259, 287, 315, and 343; *p*-values < 0.05). On study day 168, both the placebo-vaccinated control group and test-vaccinate group had an increase in titers; however, this increase occurred at nearly the same magnitude across both treatment groups suggesting the increase was assay related. In addition, titers returned to expected levels on study day 196.

### 3.5 Clinical signs of *B. bronchiseptica*

**Figure 5:**
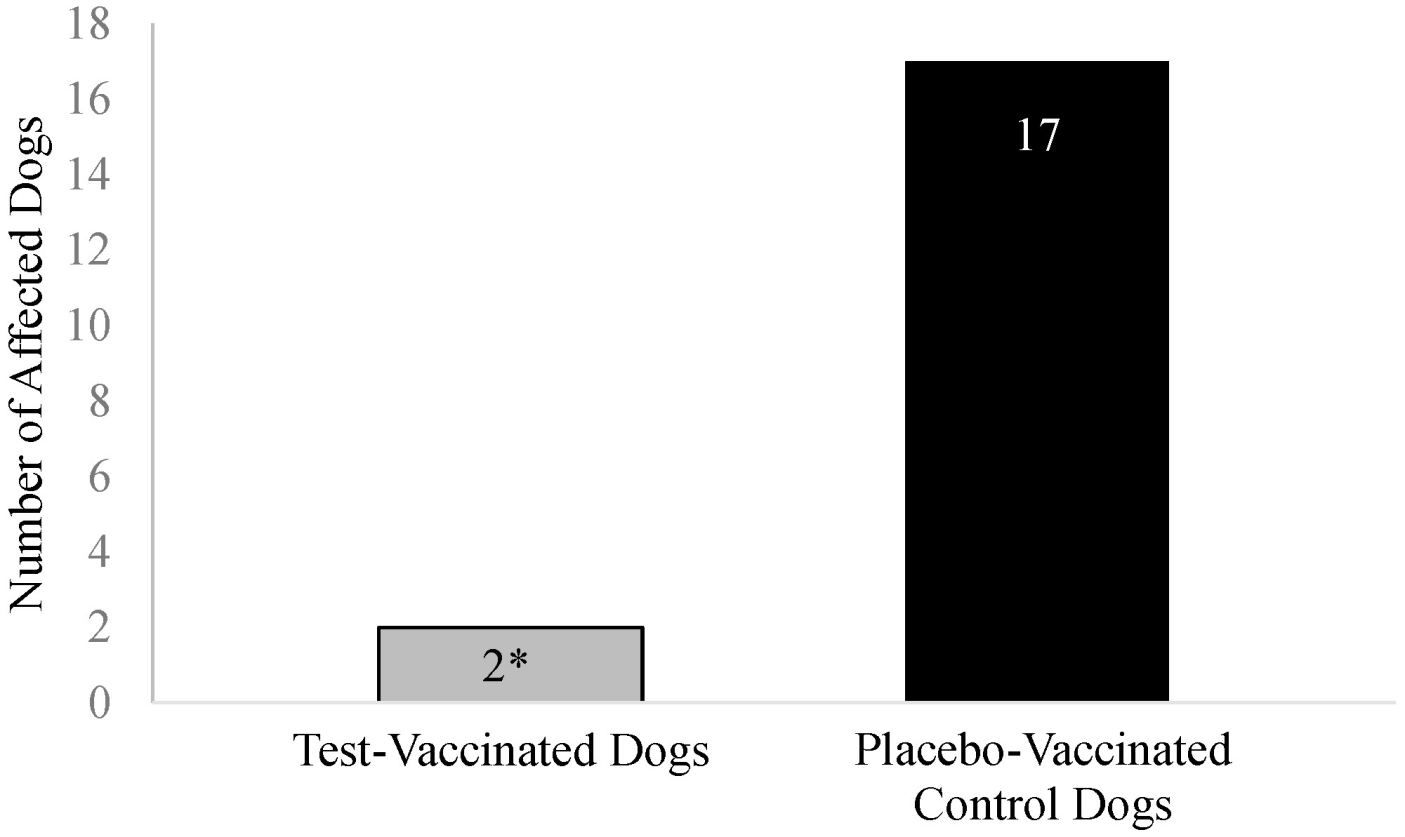
Number of *Bordetella bronchiseptica* affected dogs. The graph displays the number of *Bordetella bronchiseptica* affected dogs in each treatment group. A *B. bronchiseptica* affected dog was defined as having spontaneous coughing or spontaneous coughing with retching on 2 or more consecutive days during the post-challenge observation period. The test vaccine significantly reduced the total number of affected dogs following virulent challenge (*p < 0.0001).

**Figure 6:**
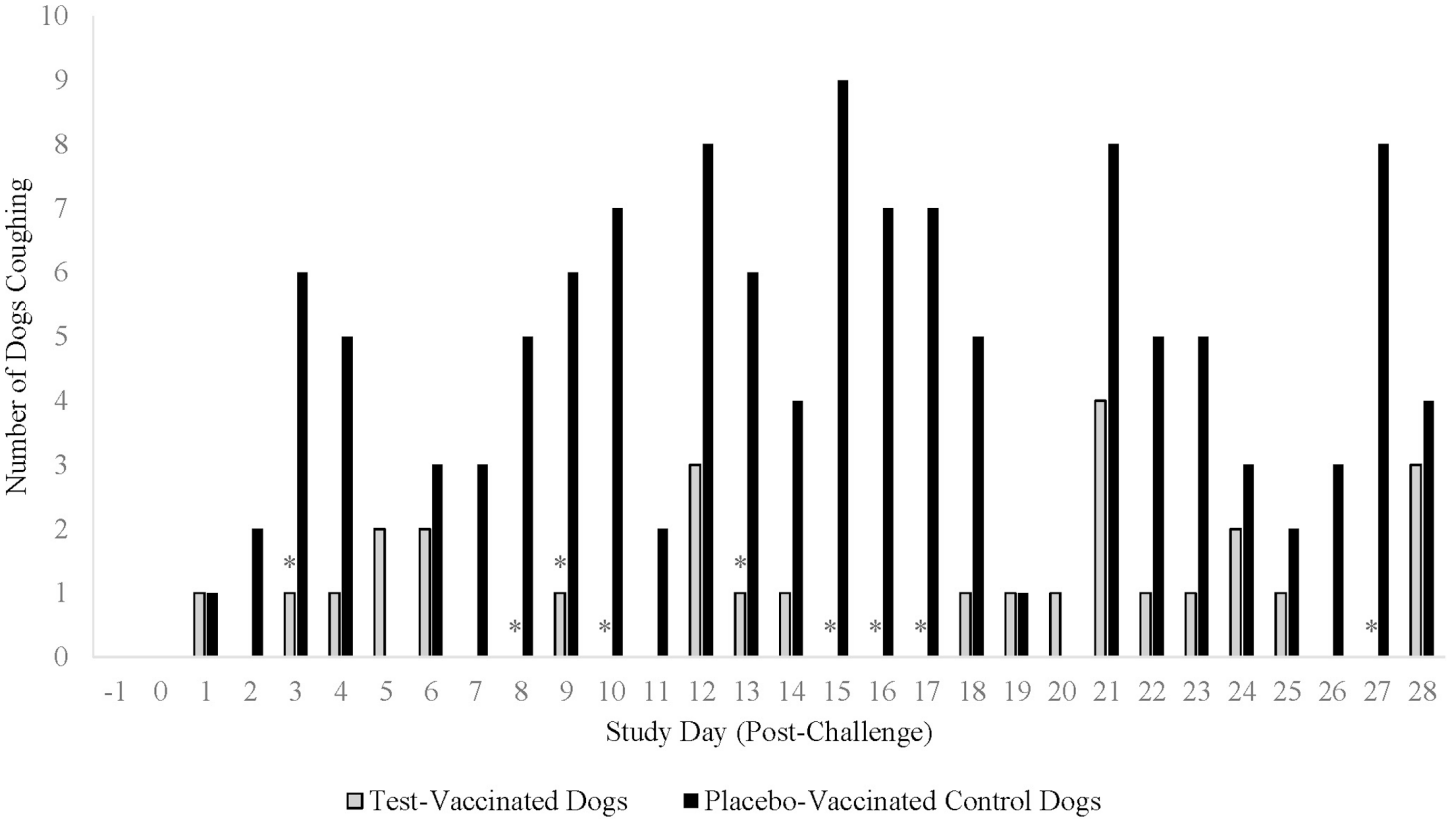
Number of dogs coughing throughout the post-challenge observation period. The graph displays the number of dogs coughing throughout the 28-day post-challenge observation period. The primary clinical sign for analysis of a *B. bronchiseptica* affected dog was spontaneous coughing or spontaneous coughing with retching. The test vaccine significantly reduced the clinical sign of coughing on 9 study days throughout the post-challenge observation period (*p ≤ 0.047).

Coughing was the primary variable to evaluate efficacy in the *B. bronchiseptica* study. An affected dog was defined as having spontaneous coughing or spontaneous coughing with retching on two or more consecutive days during the post-challenge observation period. Following challenge, 17 (74%) of the 23 placebo-vaccinated control dogs were considered affected, compared to only 2 (9%) of the 22 test-vaccinated dogs (Figure 5; *p*-value < 0.0001). In addition, the test vaccine significantly reduced the number of dogs that coughed on study days 3, 8, 9, 10, 13, 15, 16, 17, and 27 (Figure 6; *p*-values ≤ 0.047). Other clinical signs of disease such as nasal discharge, dyspnea, or depression were not observed from any of the dogs throughout the post-challenge observation period.

### 3.6 Nasal shedding of *B. bronchiseptica*

*B. bronchiseptica* was not isolated from nasal swabs prior to challenge, but following challenge, 11 (48%) of the 23 placebo-vaccinated control dogs and 10 (45%) of the 22 test-vaccinated dogs shed *B. bronchiseptica*. The test-vaccinated dogs shed less bacteria, but the difference between the groups was not significant.

## 4 Discussion

A single dose of the oral CPI + *B. bronchiseptica* vaccine provided protection against respiratory disease following challenge with virulent strains of CPI or *B. bronchiseptica* 1 year after vaccination. When comparing the test-vaccinated dogs to the placebo-vaccinated control dogs, an 83% reduction in the median duration of CPI shedding was demonstrated as well as a 65% reduction in coughing associated with a *B. bronchiseptica* infection. No systemic or local adverse events occurred in either study following vaccination. In addition to a strong safety profile, one major benefit of oral vaccination is the induction of both a strong systemic and local immune response (16). Regarding the circulating humoral immune response, both DOI studies showed strong systemic seroconversion in dogs vaccinated with the oral bivalent vaccine. Notably, seroconversion with neutralizing antibody titers to CPI occurred in 91% of dogs, and high *B. bronchiseptica* serum antibody titers occurred in 100% of dogs following one dose of the oral bivalent vaccine, respectively. Additionally, the convalescent antibody response post-CPI challenge was approximately 10-fold higher within the test-vaccinated treatment group when compared to the placebo-vaccinated treatment group likely demonstrating a strong immunologic memory response (data not shown).

Complimentarily, the local immune response was indirectly measured by assessing the ability of this oral bivalent vaccine to decrease nasal shedding of CPI following challenge. The median duration of CPI nasal shedding in dogs following challenge was significantly lower in dogs vaccinated with the oral bivalent vaccine compared to the placebo vaccine. Additionally, the viral load isolated from nasal swabs following challenge was significantly higher in the placebo-vaccinated control dogs than in the test-vaccinated dogs for 8 of the 10 days post-challenge. Furthermore, the placebo-vaccinated control dogs shed increasingly more virus from 2 to 6 days post-challenge. While there was some nasal shedding of CPI in 57% of the test-vaccinated dogs, this was not an unexpected outcome due to the robust nature of the challenge model, including comingling the dogs from both groups throughout the challenge phase of the study. Moreover, it is not unreasonable to infer that this vaccine will perform even better in the field, as dogs are typically exposed to a lower viral load and for less time than in this experimental model and dogs will receive a vaccine formulated at field dose rather than the minimum protective dose used in this study.

Similarly, the observed clinical signs of disease caused by *B. bronchiseptica* occurred in 74% of placebo-vaccinated control dogs which was significantly higher than dogs vaccinated with the oral bivalent vaccine. Furthermore, the median of the total number of days of coughing following challenge was significantly lower in dogs vaccinated with the oral bivalent vaccine compared to the placebo vaccine. Thus, the oral bivalent vaccine enabled vaccinated dogs to cough for significantly less days after challenge approximately 1 year following vaccination. Accordingly, less coughing reduces the airborne droplet formation decreasing the likelihood of environmental spread and contamination. A decrease in environmental contamination of these pathogens will ultimately lessen their magnitude for infecting mucosal surfaces of dogs in public settings. Taken together, one dose of the oral bivalent vaccine protects dogs from developing clinical signs caused from CPI and *B. bronchiseptica* and significantly decreases the amount of virus shed from the nasal mucosa as observed in these challenge models.

One limitation present in these studies was the use of purpose-bred animals for the challenge model. Using purpose-bred animals restricts assessment of vaccine performance across the inherent variability present within the general population such as multiple dog breeds, ages, health profiles, and environments. However, the test vaccine demonstrated strong protection at a minimum protective dose of antigen in purpose-bred dogs challenged with either a high load of virulent virus or bacteria one-year later. Another limitation of this study was the use of an optimized challenge model employing either a single viral or bacterial strain as the challenge organism. While this optimization allows for sound comparison between the placebo and test-vaccinated treatment groups, this impedes further evaluation of vaccine performance against other viral or bacterial strains of the same species circulating within the field. However, it should be noted that the challenge organism strains were heterologous to the vaccine strains providing additional confidence that the vaccine offers cross-protection between different viral and bacterial strains present in the field. Additionally, during each duration of immunity study as a regulatory requirement, the dogs were only challenged with one pathogen as opposed to performing a dual challenge. Despite these limitations, the data from these DOI studies demonstrates robust protection of an oral mucosal vaccine against the viral and bacterial strains used within these experimental models.

Oral mucosal vaccination is highly advantageous and can provide long lasting immunity at the site of vaccination (16). Previously published research has demonstrated mucosal vaccination methods to be efficacious for a duration of weeks to months (17). Similarly, the data from these studies demonstrate at least 12 months of protection against both CPI virus and *B. bronchiseptica*. Additional advantages of oral vaccination for dogs combine ease of use with stimulation of mucosal immunity through direct interaction with oral mucosal surfaces. A unique feature to the oral bivalent vaccine is the innovative applicator used during administration to the oral cavity that targets the nasopharyngeal tonsils allowing for greater stimulation of local immune response. During administration, the Immuno-Mist-R™ applicator creates a fine mist of vaccine capable of targeting the mucosal-associated-lymphoid-tissue (MALT) within the oral cavity contrasted with being limited to reaching the buccal pouch as observed with several other licensed oral vaccines (18). Involving the broadest area of the MALT, particularly the nasopharyngeal tonsils, is essential for induction of a strong innate immune response (19). Oral vaccination provides direct antigen interaction with MALT resulting in robust pathogen-specific mucosal immunity (20). Therefore, it is reasonable to speculate that MALT antigen-specific immunoglobulins are likely correlated with the mechanism of protection by preventing viral attachment, infection, and replication at the site of exposure and thus, subsequent shedding of virus. While this study was limited to indirect evaluation of the local immune response via measurement via nasal shedding of CPI, more research is needed to investigate the specific underlying mechanisms responsible that contribute to the success of the local mucosal immune response observed in these studies.

Effective vaccination is crucial for the prevention and reduction of clinical signs caused from a highly communicable disease such as CIRDC. Mitchell and Brownlie (17) propose that vaccines that preferentially stimulate mucosal immunity may be of greater benefit as the mucosal immune response primarily provides protection against pathogen adherence, colonization, and invasion at the mucosal surface, whereas the systemic response is more largely involved in containing and clearing infection once an infection takes hold. The results of these studies demonstrate a strong induction of systemic antibody titers coupled with a reduction in viral nasal shedding providing a multi-layered approach preventing the spread of CPI and *B. bronchiseptica*. Reduction in both viral shedding and the resulting clinical signs are vital to minimize outbreaks caused by the airborne transmission of a CPI- or *B. bronchiseptica*-infected dog, respectively. Consequently, vaccination with the oral bivalent vaccine using the Immuno-Mist-R™ applicator offers a safe and efficacious solution to combat CPI and *B. bronchiseptica* infection, remaining effective for at least 12 months.

## Data Availability

The original contributions presented in the study are included in the article/supplementary material, further inquiries can be directed to the corresponding author.
